# Synergistic effect of Ce-based nanocatalysts in the pretreatment and saccharification of raw lignocellulosic biomass: an advancement in bioethanol production

**DOI:** 10.1007/s00253-025-13627-7

**Published:** 2025-12-08

**Authors:** Mamata S. Singhvi, Chinmay Hate, Beom Soo Kim

**Affiliations:** 1 Department of Biotechnology (With Jointly Merged Institute of Bioinformatics and Biotechnology), Savitribai Phule Pune University, Pune, 411007 India; 2 Department of Chemical Engineering, Chungbuk National University, Cheongju, Chungbuk 28644 Republic of Korea

**Keywords:** Sugarcane bagasse, Bioethanol, Lignocellulosic biomass, Enzyme-mimicking nanoparticles, QRT-PCR

## Abstract

**Abstract:**

This study presents a sustainable one-step process for converting raw sugarcane bagasse (SB) into bioethanol, highlighting the innovative use of cerium-doped iron oxide nanoparticles (CeFe_3_O_4_NPs). Initially, these nanoparticles facilitated simultaneous pretreatment and hydrolysis of the raw SB biomass under ambient conditions (50 °C), demonstrating direct catalytic activity by producing 6.55 ± 0.112 g/L glucose and 4.73 ± 0.143 g/L xylose within 24 h. The scalability of this approach was confirmed with similar results achieved in a larger 7.5 L-scale fermentation. A key novelty of this research lies in demonstrating the synergistic effect of CeFe_3_O_4_NPs with enzymatic hydrolysis. By incorporating a minimal amount of in-house generated cellulase enzymes alongside CeFe_3_O_4_NPs, the sugar yields dramatically increased to 23.1 ± 1.12 g/L of glucose and 13.9 ± 0.88 g/L of xylose. This indicates that CeFe_3_O_4_NPs are not merely catalysts but function effectively as promoters, significantly enhancing the efficiency of enzymatic process. The subsequent fermentation using *Saccharomyces cerevisiae* efficiently converted these sugars, including xylose, into 17.3 ± 0.98 g/L of bioethanol with a productivity of 1.44 g/L/h. Further gene expression studies using quantitative real-time PCR (qRT-PCR) analysis revealed that CeFe_3_O_4_NPs played a role in upregulating xylose-utilizing genes within yeast strain, leading to near-complete utilization of xylose. This stimulation of xylose metabolism is a crucial finding that significantly aids in improving the overall economics of the biomass conversion process. This integrated approach, combining magnetic CeFe_3_O_4_NPs with enzymatic activity and xylose metabolism, represents a significant step towards more cost-effective and scalable bioethanol production from lignocellulosic biomass.

**Key points:**

• *Eco-friendly bioethanol production from sugarcane bagasse using nanobiotechnology*

• *Delignification and hydrolysis of biomass by enzyme-mimicking CeFe3O4 nanoparticles*

• *Xylose utilization by S. cerevisiae noticed due to CeFe3O4 nanoparticles*

**Supplementary Information:**

The online version contains supplementary material available at 10.1007/s00253-025-13627-7.

## Introduction

Conventional fossil fuels have a major limitation in their negative impact on greenhouse gas (GHG) emissions, which have caused climate change and affected human health (montgomery 2017; Rafindadi and Usman 2019). However, bioethanol derived from lignocellulosic waste is a 2nd-generation biofuel that does not affect GHG emissions and also food security. Hence, 2nd-generation biofuels proved to be a better substitute for fossil fuels (Aditiya et al. 2016). Also, the production of 2nd-generation biofuels can replace edible crops with non-food biomass like agricultural residues and wood waste. This shift reduces conflicts with food supplies and improves the sustainability of biofuel production (Duque et al. 2021). Lignocellulosic biomass (LCB) waste, which is rich in carbohydrates, is widely used to produce valuable chemicals and fuels. However, the pretreatment and enzyme hydrolysis stages are often a bottleneck in degrading. Sugarcane is a widely cultivated agricultural crop that has an annual production of 1600 million tons worldwide (Chandel et al. 2012). The two largest sugarcane-producing countries in the world are India and Brazil, accounting for almost 60% of the world’s total sugarcane production. Sugarcane bagasse (SB), a fibrous residual waste generated by sugar industries has gained attention as potential LCB substrate for biofuel production due to its composition, lower lignin content, and highly abundant nature (Ajala et al. 2021). Worldwide, approximately 0.280 billion metric tons of sugarcane waste are generated per year, which creates a significant waste disposal problem (Pan et al. 2022). SB is generated in bulk quantities by alcohol and sugar industries in India (Chandel et al. 2007), Brazil (Hernández-Salas et al. 2009), China (Cheng et al. 2008), and Indonesia(Restuti and Michaelowa 2007). Utilizing LCB waste materials such as SB for bioethanol production can help reduce disposal costs and minimize environmental issues.

Sugarcane bagasse (SB) is one mainly consisting of cellulose (40–45%), hemicellulose (30–35%), and lignin (20–30%) (Peng et al. 2009; Singhvi and Kim 2020). Owing to generation of small amount of ash content using SB substrates as compared to other LCB waste materials, it provides several advantages (Li et al. 2002). LCB is a non-edible, renewable source of fermentable and organic carbon that is suitable for high-value goods. Lignin–carbohydrate complexes (LCCs) form through covalent linkages, including esters, phenyl-glycosides, and acetyl bonds. These linkages connect lignin molecules to carbohydrate chains within plant cell walls, making the conversion of LCCs into sugars a challenging task (Feng et al. 2023). Therefore, pretreatment is necessary to break down complex linkages of LCB components to make it amenable for enzymatic hydrolysis and further produce biofuels (Singhvi and Gokhale 2019). The obtained cellulose and hemicellulose can be further hydrolyzed into simple sugars for bioethanol production through microbial fermentation.

Several pretreatment methods such as physical and chemical methods including acids (Saha et al. 2016; Sarawan et al. 2019), alkali (Lai and Idris 2016), inorganic salt (Kang et al. 2013) and ionic liquids (Auxenfans et al. 2014), and microwave irradiation (Hoang et al. 2021) have been reported. However, there are limitations to their application in biomass pretreatment due to the generation of environmentally unsafe toxic products, high cost, and the energy-intensive nature of processes (Kumar and Sharma 2017). Traditional pretreatment techniques are not enough to remove lignin and hydrogen bonds in crystalline cellulose, which results in low digestibility and high cellulase loading. Enzymatic hydrolysis for sugar production offers advantages like high selectivity and mild operating conditions, which minimize inhibitory byproducts and energy usage. However, it faces significant industrial limitations, including high enzyme costs, long reaction times, and poor stability against temperature and pH changes. Additionally, enzymes suffer from product inhibition and mass transfer issues at high substrate concentrations, which hamper efficiency. It is important to find sustainable and energy-efficient ways to pretreat LCB materials (Lyu et al. 2019). Magnetic nanoparticles, such as CeFe_3_O_4_NPs, provide a robust alternative due to their superior thermal and chemical stability, reusability facilitated by magnetic separation, and cost-effectiveness. These nanocatalysts often possess higher surface areas for increased efficiency and can mimic multi-enzyme functions. Studies on nanocatalysts have demonstrated their potential in biomass hydrolysis and have shown that iron oxide nanoparticles can even stimulate bioethanol fermentation. Nanomaterials offer numerous advantages, such as economical synthesis, better stability, and enhanced catalytic capacity as compared to microbial enzymes. As a result, they are being extensively studied for various applications in fields like polluted water treatment, pharmaceuticals, biosensors, conversion of LCB materials, and in the production biochemicals and biofuels (Hokkanen et al. 2016; Zhou et al. 2017; Singhvi et al. 2021a, b; Rajak et al. 2021; Singhvi et al. 2022). Nanotechnology has opened up new possibilities for researchers to search the use of various nanomaterials like Fe_3_O_4_, CeO_2_, AuNPs, etc., in enzyme-like activities such as oxidase and peroxidase (Singh 2016; Zhang et al. 2018). Fe-based NPs possess unique enzyme-mimetic properties which can be modified by altering their morphology and size and by doping them on nanomaterials (Liu and Liu 2017). Researchers have investigated the action of zeolite-supported nickel and molybdenum oxide carbon nanotubes (MoOx/CNT) on degradation of lignin compounds (Xiao et al. 2017). Cerium-based nanomaterials have shown peroxidase-mimicking activity which can be applied in LCB conversion process as reported earlier (Kim et al. 2023).

Cerium-based nanomaterials have become increasingly popular because of their exceptional properties, such as better oxygen mobility, excellent thermostability, and impressive reducibility. The reducibility is related to the formation of either apparent oxygen vacancies or rapid modifications between Ce^3+^/Ce^4+^ species (Cargnello et al. 2013). In addition, the catalytic performance of Fe_3_O_4_ NPs has been improved by doping it with cerium (Rajak et al. 2021). This is due to the synergistic effect between Fe + and Ce^3+^/Ce^4+^ that enhances its bifunctional exchanges. CeFe_3_O_4_ NPs are active oxygen pumps that swiftly donate their framework oxygen atoms for oxidation reactions. The reduced CeFe_3_O_4_ NPs are then re-oxidized by O_2_ facilitating the transport of O_2_, which can help in the deconstruction of recalcitrant LCB materials (Kharton et al. 2003; Zhang et al. 2025). The current research investigates the application of CeFe_3_O_4_NPs for sugar hydrolysis and fermentation, positioning them as a promising solution to address the persistent challenges of enzymatic methods.

The accomplishment of bioconversion process relies on the methods used during pre-treatment, hydrolysis, and fermentation stages. The cost incurred in each step of the bioconversion process has a direct impact on the feasibility of cellulosic ethanol (Aui et al. 2021). The lignocellulose biorefinery has immense potential, but it requires focused research to make the most of the lignocellulosic biomass without any wastage. Pretreatment of LCB materials and enzyme production are important steps in the LCB conversion process which contribute majorly in the overall process cost (Cheng et al. 2019; Aui et al. 2021). Our team has developed a novel biorefinery process that eliminates the pretreatment step and uses a small amount of enzyme preparations made from cheaper carbon substrates. For our study, we used raw sugarcane bagasse (SB) as a biomass substrate for bioethanol fermentation. SB is an ideal choice for agricultural waste due to the large-scale cultivation of sugarcane in India. LCB materials hydrolysis is a process that has traditionally been carried out using enzymes produced by microbes. However, the use of these enzymes is limited due to their high production costs and susceptibility to damage. Therefore, researchers have been working on developing artificial materials that can mimic the catalytic function of natural enzymes (Singhvi et al. 2021b; Rajak et al. 2021). A recent study has found that CeFe_3_O_4_ NPs have similar reactivity as oxidase and cellulase–hemicellulase enzymes (CHE).

A large-scale technology for producing microbial ethanol has not been established till date. To address this, a bioprocess in 7.5-L fermenter level has been developed that can simultaneously delignify and saccharify raw SB biomass substrates in a single step. This process uses CeFe_3_O_4_NPs to generate fermentable sugars. By adding a small amount of in-house-produced CHE preparations during the simultaneous pretreatment and hydrolysis (SPH) process, a maximum of 23.1 ± 1.12 g/L of glucose and 13.9 ± 0.88 g/L of xylose sugars were released which were further diverted to produce ethanol by *Saccharomyces cerevisiae*. In this study, we used a different method to synthesize CeFe_3_O_4_ NPs, which resulted in different chemical, physical, and functional properties as compared to previously reported studies (Rajak et al. 2021). Ultimately, this strategy can offer an eco-friendly greener process that is suitable for converting LCB to bioethanol on a large scale.

This investigation explores the synergistic action between CeFe_3_O_4_ nanoparticles (NPs) and cellulase–hemicellulase enzymes for enhanced lignocellulosic biomass (LCB) conversion. The synthesized NPs exhibit intrinsic oxidase- and cellulase–hemicellulase-mimicking activities. These properties enable efficient lignin removal and subsequent polysaccharide hydrolysis. The central objective of utilizing these NPs is to overcome the recalcitrance of LCB by replacing traditional, energy-intensive, and costly pretreatment methods. In our study, we leveraged in-house-produced cellulase–hemicellulase enzymes to hydrolyze the delignified LCB, which resulted in a substantial release of fermentable sugars. A combined simultaneous pretreatment and hydrolysis (SPH) process was also developed to assess the combined effect of the NPs and enzymes. This integrated approach revealed a significant synergistic effect, wherein the NPs enhanced the catalytic efficiency of the enzymes. By completely eliminating the conventional pretreatment stage and reducing the required enzyme concentration, this process offers a highly cost-effective strategy for producing fermentable sugars from LCB.

## Materials and methods

### Materials, chemicals, and reagents

Sugarcane bagasse (SB) biomass substrate was obtained from local juice shops in Pune, India, and dried at 50–60 °C. The dried SB substrate was finely ground in grinder to produce a powder with particle size of about 0.2 mm, which was then utilized in biomass degradation studies. Cerium nitrate (Ce(NO_3_)_3_·6H_2_O), acetic acid (> 99.0%), ammonia solution (25%), iron chloride hexahydrate (FeCl_3_·6H_2_O), and ferrous sulfate hexahydrate ((FeSO_4_.6H_2_O) were obtained from Himedia company, India. Cellulose powder, carboxymethyl cellulose (CMC), *p*-nitrophenyl-β-D-glucopyranoside (*p-*NPG), dinitrosalicylic acid (DNS), n,o-bis(trimethylsilyl) trifluoroacetamide, tetramethyl-p-phenylenediamine dihydrochloride (TMPD), and laccase produced by *Trametes versicolor* were obtained from Sigma-Aldrich, USA. All other reagents and chemicals were purchased locally.

### Cerium-based Fe3O4 nanoparticles (CeFe3O4 NPs) synthesis and its characterization studies

The synthesis of CeFe_3_O_4_NPs was carried out using hydrothermal method as previously reported by Kim et al. (Kim et al. 2023) with slight modifications. Instead of using higher temperature and longer duration conditions, we kept the reaction mixture for autoclaving for 20 min. An aqueous solution of FeCl_3_·6H_2_O and FeSO_4_.6H_2_O was prepared by dissolving 0.540 g and 0.278 g, respectively, in 100 mL of deionized water. The solution was stirred for 5 min at room temperature. A 20-mL volume of 25% aqueous ammonia was subsequently added dropwise to the mixture under continuous stirring. Following this, cerium nitrate hexahydrate (Ce(NO_3_)_3_·6H_2_O, was added, with concentrations varying between 3.0 wt%, dispersed in 100 mL of deionized water. The resulting mixture was then autoclaved for 30 min. After cooling the mixture, final precipitate was magnetically recovered and purified by washing multiple times with deionized water, followed by a final wash with ethanol. The purified product was dried in an oven at 60 °C.

The synthesized CeFe_3_O_4_NPs were confirmed using various techniques as reported earlier (Singhvi et al. 2021b; Rajak et al. 2021), including transmission electron microscopy (TEM) and scanning electron microscopy (SEM) analysis to estimate the morphology and size of the NPs. The elemental composition of the synthesized NPs was analyzed using energy-dispersive X-ray spectroscopy (EDS) technique. Fourier transform infrared (FTIR) analysis was performed using Shimadzu: IR Affinity 1S to detect the presence of functional groups on synthesized NPs. Zeta potential and particle size and of NPs were estimated using Zetasizer (ZS90 Nano Series, Malvern Instrument). CeFe_3_O_4_NPs were sonicated for about 10–15 min for appropriate distribution.

### Microorganism, media, and enzyme production

A fungal strain called *Penicillium janthinellum* NCIM 1171, obtained from NCIM Resource Centre, Council of Scientific and Industrial Research-National Chemical Laboratory (CSIR-NCL) in Pune, India, was used to produce cellulase and hemicellulase enzymes (CHE). The strain was regularly maintained on potato dextrose agar (PDA) slants and sub-cultured after every 3 months.

### Enzyme production at flask level

CHE production experiments were conducted in 250-mL shake flasks with a 70 mL basal medium (BM) (MANDELS and WEBER 1969) containing 2.5% (w/v) of different LCB substrates and 1.0% cellulose powder as optimized previously by Adsul et al. (Adsul et al. 2007). Various LCB substrates including wheat bran (WB), corn cob (CC), sugarcane bagasse (SB), and soya husk (SH) along with cellulose (1%) were used as carbon source in BM for CHE production under submerged conditions as reported earlier (Adsul et al. 2007; Singhvi et al. 2011, 2022). The flasks were inoculated with spores (approximately 10^7^) from *P. janthinellum* grown PDA slant and incubated at 28 °C with shaking at 150 rpm. Every 2 days, 2-mL samples were taken and centrifuged at 10,000 rpm for 10 min. The obtained supernatant samples were analyzed for extracellular enzyme activities, especially cellulase and hemicellulase. The enzyme supernatant was then used for raw SB substrate hydrolysis at the flask level.

#### Enzyme production at 7.5-L fermenter scale

CHE production was carried out in a 7.5-L fermenter with water-jacketed vessels made by RALF Bioengineering, Inc. (Massachusetts, USA) with the working volume of 4.5 L. SB was used as a carbon source, along with cellulose powder as an inducer in the BM, as mentioned in the previous section. The pH was maintained between 4.0 and 5.0 using 2 M HCl and NaOH. Aeration was adjusted to about 0.8 VVM (volume of sparged air to volume of liquid per minute) compressed air, and DO above 20% with an agitation cascade of 300–700 rpm. The fermenter was inoculated with a 2.5% (v/v) inoculum of 4-day grown culture of *P. janthinellum* strain in the same fermentation batch medium as mentioned in the “Enzyme production at flask level” section. Samples were withdrawn every 24 h, and the obtained supernatants were stored at − 20 °C for further analysis after being centrifuged at 12,000 rpm for 5 min. After optimizing the conditions, the complete fermented broth was removed after the second day of fermentation and centrifuged, and the obtained supernatant was used for hydrolysis experiments. The bioreactor was terminated once DO showed a constant ascending tendency for more than 8 h, indicating starvation of cells.

### Enzyme activity determination

#### Cellulase–hemicellulase activity determination of enzyme supernatant and CeFe_3_O_4_ NPs.

The enzyme supernatant and CeFe_3_O_4_NPs were tested for both cellulase and hemicellulase enzyme activities as per reported previously (Singhvi et al 2011). Cellulase enzyme including exoglucanase (FPase), endoglucanase (CMCase), and β-glucosidase activities determined using Whatman no.1 filter paper (25 mm), carboxymethyl cellulose (CMC), and *p-*NPG substrates respectively. In case of hemicellulase activity determination, xylan (from beechwood, Megazyme) substrate was used. All enzyme assays were conducted at 50 °C in sodium citrate buffer (50 mM, pH 4.5) as reported earlier (Singhvi et al. 2011; Nagraj et al. 2014).

#### Enzyme-mimicking activities of CeFe_3_O_4_ NPs

Oxidase activity was determined in an assay system of total reaction mixture containing 0.80 mL of citrate buffer (50 mM, pH 4.5), 0.60 mL of 3,3′,5,5′-tetramethyLCBenzidine (TMB) (1 mg/mL) solution, and 0.10 mL NP solution (4 g/L). The reaction mixture was incubated at 37 °C for half an hour, and after incubation, the absorbance was taken at a wavelength of 420 nm (Hosseini et al. 2017; Wu et al. 2019).

### Simultaneous pretreatment and hydrolysis (SPH) process at flask- and 7.5-L fermenter scale

In this experiment, synthesized nanoparticles (NPs) were used to pre-treat raw sugarcane bagasse (SB) biomass substrate. At the same time, submerged cellulase and hemicellulase enzymes derived from submerged fermentation were added to enhance the saccharification of the raw SB. Firstly, experiments were performed in flasks containing citrate buffer (50 mM, pH 4.5) with varying concentrations of SB biomass substrate (5.0%, 10.0%, and 20.0% w/v), CeFe_3_O_4_ NPs (2.0 wt%), and in-house produced enzymes (5.0 FPU/g of substrate). A control flask without CeFe_3_O_4_ NPs and enzymes was also included. The SPH reaction was performed at 50 °C with shaking at 150 rpm.

Further, SPH process was conducted on a larger scale with 5.0 L volume of reaction mixture containing SB substrate concentration of 20.0% (w/v), 5 FPU of enzyme preparations/g of substrate, and CeFe_3_O_4_NPs at a concentration of 2.0 wt% of total substrate used. The SPH process was carried out at 50 °C for 24 h with shaking at 150 rpm. Afterward, the hydrolysate was separated from the biomass material using a muslin cloth. The resulting supernatant was analyzed for glucose, xylose, and total reducing sugar (TRS) contents, and was used for bioethanol fermentation with *S. cerevisiae* yeast strain.

### Bioethanol fermentation at flask- and 7.5-L fermenter scale

*Saccharomyces cerevisiae* (KCTC 7928) yeast was grown on YM plates consisting of (per L): 3 g yeast extract, 3 g malt extract, 5 g peptone, 10 g glucose, and 20 g agar incubated at 30 °C for 24 h and then stored at 4 °C following a previously reported method (Zheng et al. 2019). Inoculum preparation was done by inoculating *S. cerevisiae* in YM media and incubating it at 30 °C with shaking at 150 rpm. For the fermentation process, a 12-h-old culture of *S. cerevisiae* (5%) cells were inoculated into stoppered flasks containing hydrolysate obtained from 20% of raw SB (w/v) during SPH process. Nitrogen sources, including yeast extract (3.0 g/L), malt extract (3.0 g/L), and peptone (5.0 g/L), were added along with hydrolysate and autoclaved. Ethanol fermentation was conducted in 250-mL stoppered flasks at 30 °C with shaking at 150 rpm for 24 h using 80 mL of media. Samples were removed at different time intervals, and the supernatant was analyzed for sugar and ethanol contents during fermentation.

Further, experiments were carried out for larger-scale bioethanol fermentation using a fermenter that had a working capacity of 5.0 L (RALF Bioengineering, Inc. in the state of Massachusetts, USA). The pH level was maintained between 5.0 and 6.0 by using 2 M HCl and NaOH. Aeration was adjusted to approximately 0.6 VVM compressed air, and DO was maintained above 20% with an agitation cascade of 300 rpm. The total volume of the fermenter was 5.0 L, and hydrolysate obtained from SB substrate (20% w/v) was inoculated with 5% (v/v) inoculum of 12–16-h grown culture of *S. cerevisiae* strain in the same fermentation medium as mentioned above. Samples were taken every 3 h, centrifuged at 10,000 RPM for 5 min, and the obtained supernatants were stored at − 20 °C for further analysis. The bioreactor was terminated after 24 h when DO showed a constant descendant tendency for more than 4 h, indicating starvation of cells. High-performance liquid chromatography (HPLC) analysis was performed for the determination of glucose, xylose, and ethanol concentrations in the fermented broth samples. The TRS concentration was estimated using DNS method (Miller 1959).

### RNA isolation and quantitative one-step real-time PCR (qRT-PCR) using xylose fermenting gene-specific primers

The total RNA was extracted from the cells as described by Singhvi et al. (Singhvi et al. 2017) with slight modifications.Cell pellets of *S. cerevisiae* grown in control (laccase-treated SB, LSB) and test (CeFe_3O4_ NP-treated raw SB, RSB) samples were suspended in 100 mL of solution A, which contained 20 mM sodium acetate, 1 mM EDTA, and 0.5% SDS (pH 5.0), along with phenol saturated with sodium acetate. The mixture was incubated at 65 °C with shaking for 10 min and then centrifuged at 12,000 rpm for 5 min. The upper aqueous phase was transferred to a new tube. Next, 150 mL of cold 100% ethanol was added, mixed, and centrifuged again at 12,000 rpm for 5 min. The supernatant was discarded, and 200 mL of cold 70% ethanol was used to rinse the pellet, followed by a centrifugation for 30 s. The RNA pellet was suspended in 50 mL of RNase-free water. To remove genomic DNA, samples were treated with DNase I (Takara Bio Inc.) for 30 min at 37 °C and stored at − 20 °C. RNA integrity was verified using 2% agarose gel electrophoresis, and concentration and purity were measured at 260 nm with a NanoDrop spectrophotometer. The purified RNA was then analyzed using quantitative real-time PCR (qRT-PCR) to quantify xylose-utilizing gene expression.

Real-time PCR was carried out using the One-Step SYBR PrimeScript RT-PCR Kit II (Takara, Japan) on a Bio-Rad Cycler instrument in 96-well plates. One-Step SYBR PrimeScript RT-PCR Kit II performed cDNA synthesis from RNA using PrimeScript Reverse Transcriptase and PCR amplification with DNA polymerase within one tube continuously. PCR amplification products were then monitored in real-time using SYBR Green I detection. Reverse transcription was carried out in a condition of 5 min at 42 °C and 10 s at 95 °C, and PCR reaction was performed in 40 cycles of 10 s at 95 °C, 30 s at 58 °C, and 30 s at 72 °C. The primers used for qRT-PCR verification were designed by using NCBI Primer blast software based on the available DNA sequences as given in Table S1. The relative quantification (2^−∆∆CT^) method was used to determine the target gene, xylitol dehydrogenase (XDH), xylose reductase (XR), and xylulokinase (XK) levels, and ACT 1 and RDN 18 were used as reference genes. *S. cerevisiae* cells grown in media containing laccase*-*treated SB (LSB) without NPs were considered as the control, and the cells grown in media containing raw SB (RSB) along with the addition of CeFe_3_O_4_ NPs were referred as test. From these results, a ratio of the concentration of gene-specific mRNA in the sample was calculated. The data obtained in the form of* C*_T_ value were analyzed using the comparative critical threshold (∆∆*C*_T_), and relative expression of genes were calculated as reported earlier (Singhvi et al. 2017).

### Recycling of CeFe_3_O_4_ NPs used for SB hydrolysis

After the SPH process, CeFe_3_O_4_ NPs attached to solid SB biomass residue was separated out by applying a magnetic field, then washed with water and kept for overnight drying at 50 °C. These NPs were reused for SPH experiments to test the efficacy of used NPs. SPH experiments were carried out by using the same method as mentioned in the above section.

### Characterization of untreated and treated SB samples

Both untreated and treated SB samples were evaluated for the structural and functional changes that might have occurred after SPH process was applied to the raw SB biomass. To investigate the changes in the peripheral morphology of the raw SB samples, SEM analysis was conducted. Samples were coated with platinum and analyzed using a voltage of 3 kV. Further, FTIR was performed to detect functional groups and absorption peaks of samples as reported earlier (Singhvi et al. 2021b). For the comparison of crystallinity of both untreated and treated SB samples, XRD (X’Pert-Pro, PANalytical) was used using the same conditions as reported earlier (Rajak et al. 2021) The crystallinity index (CrI %) and crystallite size (D) of both samples were estimated as reported previously (Gurgel et al. 2012), (Zhu et al. 2011). Porosity (including pore size and volume, and surface area) of both SB samples were scrutinized using BET surface area and pore size analyzer (BELSORP MR1,Microtrac). Both the samples were kept in degassing module for 3 h at room temperature. Surface area of both samples was calculated using Brunauer–Emmett–Teller (BET) method, and pore diameter and volume were estimated using Barrett-Joyner-Halenda (BJH) method (Brunauer et al. 1938;  Barret et al. 1951).

### Analytical methods

Both untreated and treated SB samples were examined for biochemical composition using the standard method as reported earlier (Ayeni et al. 2015). During SPH process, the released phenolic content was assessed using Folin–Ciocalteau method (Singleton et al. 1999). Glucose, xylose, and ethanol concentrations were analyzed using HPLC (Shimadzu). The fermented broth samples obtained after SPH process and ethanol fermentation were analyzed by injecting into a Aminex hpx-87 h column (Bio-Rad) at 50 °C with a refractive index (RI) detector. All HPLC conditions used were as per reported earlier (Singhvi et al. 2021b). All the samples obtained during SPH process were analyzed using gas chromatography-mass spectrometry (GC–MS) so as to understand the action of CeFe_3_O_4_ NPs on lignin degradation. GC–MS analysis was performed through Shimadzu TQ 8030 GC equipped with detector using the same conditions as reported earlier (Rajak et al. 2021).

## Results

### Physical and chemical characterization of CeFe_3_O_4 _NPs

**Fig. 1:**
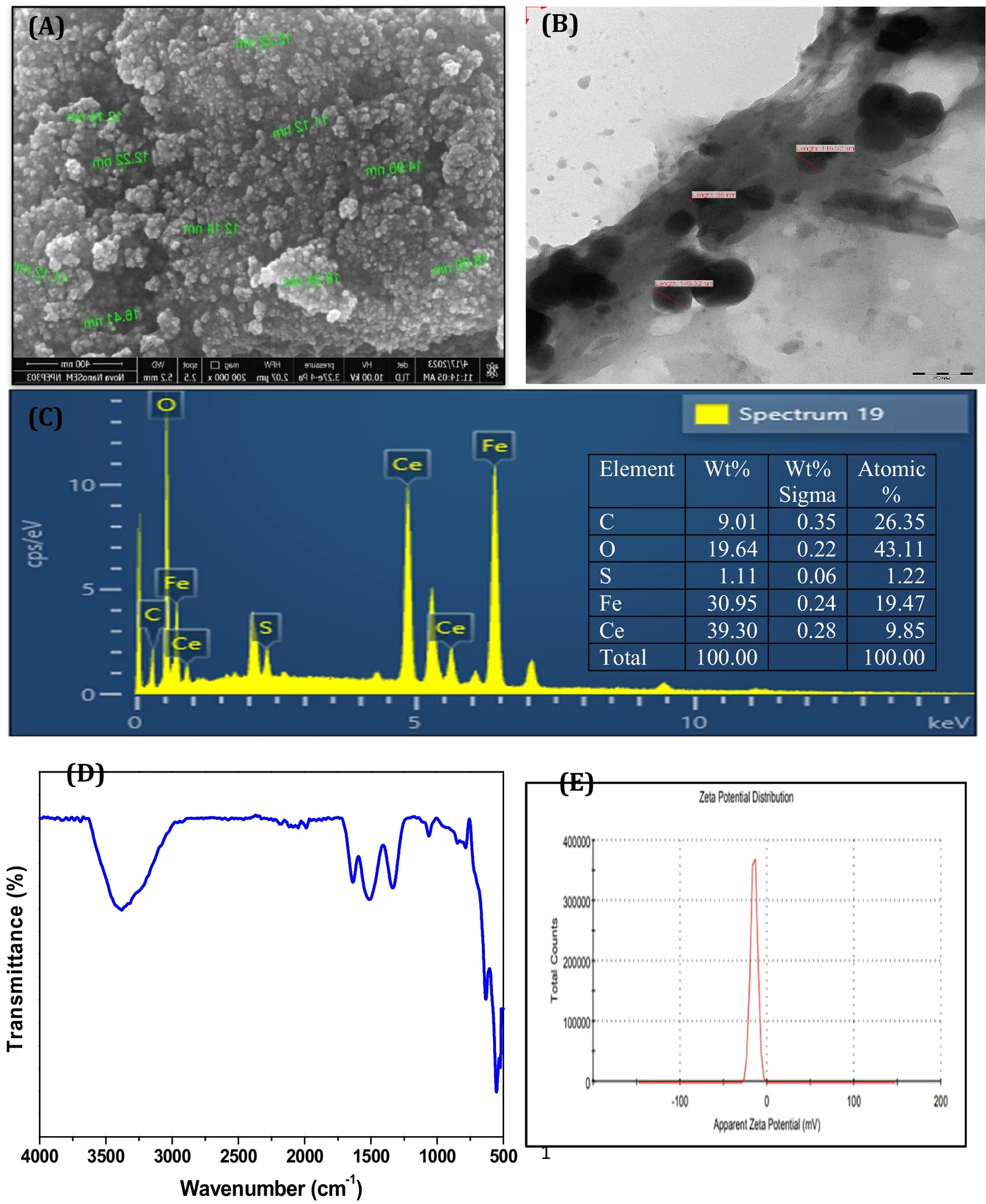
Characterization of CeFe_3_O_4_-NPs using(**A** SEM, **B** TEM, **C** EDS, **D** FTIR, and **E** zeta potential analysis

The synthesis of CeFe_3_O_4_NPs using modified method was confirmed by using SEM, TEM EDS, FTIR, and DLS analysis. Additionally, CeFe_3_O_4_ NPs were characterized using XRD and TGA. To comprehend the physical modifications, the size and morphology of CeFe_3_O_4_NPs were examined through TEM and SEM analysis which demonstrated agglomerated sphere-shaped particles with a size ranging from 11 to 16 nm (Aashima et al. 2019) (Fig. 1A and B). Figure 1C represents the elemental analysis of CeFe_3_O_4_NPs using EDS technique. The presence of peak at 4.75 and 6.5 keV corroborates the occurrence of cerium (39.30%) and iron (30.95%) elements substantiating the higher content of cerium than iron. Also, FTIR analysis was carried out to identify the functional groups present in NPs displaying individual peaks at 515 cm^−1^ and 585 cm^−1^ wavelength corresponding to Ce–O and Fe–O groups. The presence of absorption peaks at 1510, 1645, and 3470 cm^−1^ ascribing to vibration of –NH, C = O and –OH groups, correspondingly, validate the conjugation of synthesized CeFe_3_O_4_NPs (Aashima et al. 2019; Rajak et al. 2021) (Fig. 1D). DLS analysis was conducted to investigate zeta potential (ZP) and hydrodynamic size of CeFe_3_O_4_NPs. The zeta potential (ZP) studies of the synthesized NPs are significant factor for understanding the stability and surface chemistry of NPs in the colloidal phase. As shown in Fig. 1E and Table S2, the measured ZP value was − 15.0 ± 3.92 mV, which demonstrates that NPs have a net negative charge on its surface and are stable. Further, XRD analysis was performed to check crystallinity and thermal stability of synthesized NPs. XRD analysis of the polycrystalline samples divulges the formation of a definite single-phase inverse spinel structure. As shown in Fig. S1, XRD analysis of NPs exhibited a series of characteristic peaks of pure Fe_3_O_4_ (2θ = 30.3, 35.6, 43.5, 53.7, 57.2, and 62.7) corresponding to the 220, 311, 400, 422, 511, and 440 planes (Aashima et al. 2019). It also specifies that agglomeration persists due to the magnetic property of Fe_3_O_4_. (Fig. S1). The magnetic properties of CeFe_3_O_4_NPs were tested by applying magnetic field as shown in Fig. S2. Even after the doping of cerium, Fe_3_O_4_ still possessed its ferromagnetic properties, which is beneficial for separation of nanomaterials easily from the hydrolysate in the presence of magnetic field.

### Enzyme production using sugarcane bagasse as a carbon source at 7.5-L scale

In this study, several substrates such as wheat bran (WB), corn cob (CC), sugarcane bagasse (SB), and soya husk (SH) have been evaluated. Among all the substrates used, SB source found to be a better LCB residue as *P. janthinellum* secreted higher concentrations of extracellular protein using SB as a carbon source (as shown in Table S3). *Penicillium janthinellum* NCIM 1171 was evaluated for extracellular cellulase and hemicellulase production in shake flask containing BM along with 4.0 g of SB substrate and 1.0 g of cellulose under submerged conditions (Table 1). Table 1 displays a comparative profile of CHE secretion on SB substrate both at flask- and fermenter-level.

**Table 1:** Cellulase and hemicellulase enzyme production by *P. Janthinellum* NCIM 1171 under submerged fermentation conditions at flask- and fermenter-level

Scale	Fermentation time (day)	Enzyme activities (IU/mL)				Hemicellulase
		Cellulase				
		Exoglucanase		Endoglucanase	β-glucosidase	Xylanase
Control*	**2nd**	0.004 ± 0.0001		0.391 ± 0.012	0.321 ± 0.011	0.227 ± 0.013
	**4th**	0.022 ± 0.0001		0.542 ± 0.028	0.416 ± 0.012	0.286 ± 0.015
	**6th**	0.032 ± 0.0012		0.980 ± 0.051	0.788 ± 0.028	0.438 ± 0.011
	**8th**	0.041 ± 0.0021		1.000 ± 0.043	0.884 ± 0.031	0.491 ± 0.021
Flask	**2nd**	0.143 ± 0.010		1.961 ± 0.142	1.081 ± 0.009	0.587 ± 0.031
	**4th**	0.222 ± 0.012		2.842 ± 0.180	2.864 ± 0.125	1.860 ± 0.081
	**6th**	0.427 ± 0.018		3.604 ± 0.201	3.783 ± 0.152	2.268 ± 0.123
	**8th**	0.391 ± 0.031		4.007 ± 0.231	3.243 ± 0.132	1.991 ± 0.112
Fermenter	**1st**	0.198 ± 0.008		2.496 ± 0.124	1.281 ± 0.009	0.487 ± 0.022
	**2nd**	0.328 ± 0.015		3.942 ± 0.210	3.994 ± 0.125	1.990 ± 0.079
	**3rd**	0.337 ± 0.012		3.981 ± 0.199	4.073 ± 0.152	3.248 ± 0.147
	**4th**	0.221 ± 0.013	3.877 ± 0.221		3.543 ± 0.132	2.791 ± 0.142

Control* experiments contained only 1% cellulose without any SB substrate in BM media. Fermentation experiments were carried out at 30 °C under submerged conditions in fermentation medium containing sugarcane bagasse (2.5%) and cellulose (1.0%) substrates. The standard deviation values represented in the table are derived from the experiment performed in triplicates

In case of flask-level experiments, the highest CHE activities were obtained on the 6th day of fermentation except for endoglucanase activity which was maximum on the 8th day of fermentation. *P. janthinellum* demonstrated maximum activities of exoglucanase (0.427 ± 0.018 IU/mL), β-glucosidase (3.783 ± 0.152 IU/mL), and xylanase (2.268 ± 0.123 IU/mL) enzymes on the 6th day; and maximum endoglucanase activity (4.007 ± 0.231 IU/mL) was observed on the 8th day ((as shown in Table 1) in the BM containing SB (2.5%) and cellulose (1%) under submerged fermentation conditions. All enzyme activities were decreased on the 8th day of fermentation. At fermenter-level, higher activities of exoglucanase (0.337 ± 0.012 IU/mL), endoglucanase (3.981 ± 0.199 IU/mL), β-glucosidase (4.073 ± 0.152 IU/mL), and xylanase (3.248 ± 0.147 IU/mL) were achieved on the 3rd day of fermentation (as shown in Table 1), demonstrating a reduced time as compared to the flask level enzyme production.

### Enzyme-mimicking activities of CeFe_3_O_4 _NPs

CeFe_3_O_4_NPs were tested for cellulase–hemicellulase and oxidase-mimicking activities by using the standard assay as described in the “Enzyme activity determination” method section. The synthesized NPs exhibited cellulase–hemicellulase activities as displayed in Table 2. Both cellulase and hemicellulase activities of CeFe_3_O_4_NPs were determined as per the protocol reported earlier (Singhvi et al. 2011). Cellulase enzymes are cocktail of endoglucanase (CMCase), exoglucanase (FPase), and β-glucosidase (Cellobiase) enzymes which act sequentially/synergistically during cellulose depolymerization. Endoglucanase randomly cleaves β−1,4 linkages of the cellulose chains to release free ends, which are further splitted into 2–4 units by the action of exoglucanase to generate oligosaccharides, which are lastly hydrolyzed by β-glucosidase into glucose molecules (De Paula et al. 2019). Furthermore, hemicellulase enzymes can breakdown the hemicellulose biomass component to increase the accessibility of cellulose for hydrolysis (Sanhueza et al. 2018). Table 2 displays cellulase- and hemicellulase-mimicking activities of CeFe_3_O_4_NPs. Among cellulases, β-glucosidase (0.211 ± 0.009 IU/mg) exhibited higher activity than exoglucanase (0.059 ± 0.0023 IU/mg) and endoglucanase (0.029 ± 0.0011 IU/mg). Also, CeFe_3_O_4_NPs revealed hemicellulase activities (0.073 ± 0.0035 IU/mg) when xylan was used as a substrate. The application of CeFe_3_O_4_NPs unveiling cellulase- and hemicellulase-mimicking activities can be a sustainable and economical option to costly pretreatment and enzymatic hydrolysis processes required for biomass conversion.

**Table 2:** Determination of cellulase–hemicellulase and oxidase activities of CeFe_3_O_4_NPs

Oxidase activity (chemical oxidation) (IU/mg)		Cellulase activities (IU/mg)		Hemicellulase activity (IU/mg)
0.3407 ± 0.051	**Exoglucanase**	**Endoglucanase**	**β-glucosidase**	
	0.059 ± 0.0023	0.029 ± 0.0011	0.211 ± 0.0009	0.073 ± 0.0035

**Fig. 2:**
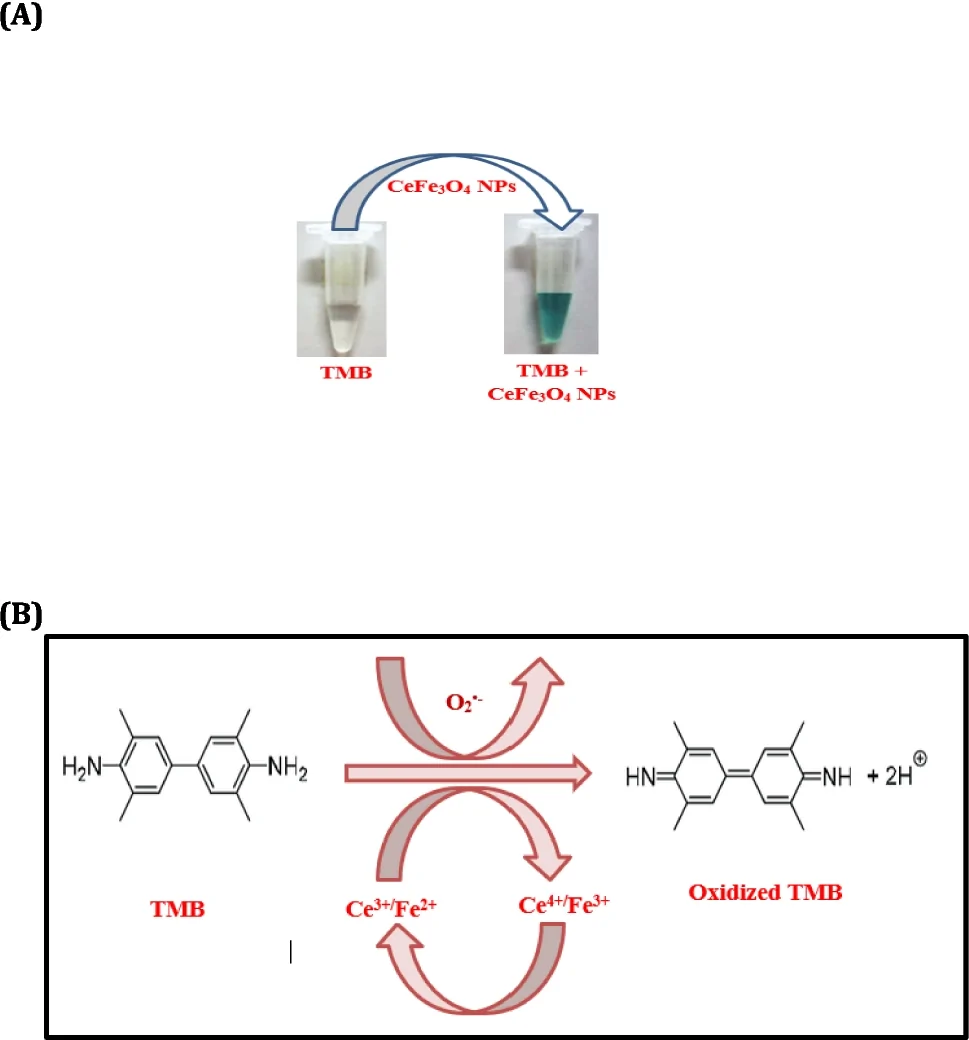
Oxidase activity of synthesized CeFe_3_O_4_NPs due to chemical oxidation. **a** Change in color of substrate in the presence of CeFe_3_O_4_NPs due to TMB oxidation in the assay mixture. **b** The probable mechanism of the oxidase mimicking behavior of CeFe_3_O_4_ NPs

The chemical oxidation activity of CeFe_3_O_4_ NPs is shown in Fig. 2A and B. The oxidized TMB in the mixture was detected by a color change from colorless to bluish green and its absorbance peak at 652 nm. By using the absorbance value, oxidase activity of NPs was calculated as 0.3407 ± 0.051 IU/mg. The oxidase activity of CeFe_3_O_4_ NPs was higher in this study than previously synthesized NPs (Rajak et al. 2021) owing to higher percentage of cerium. It is widely known that CeONP has a greater attraction for TMB than H_2_O_2_, owing to its oxidase property.

1 $${\mathrm{Ce}}^{4+}+{\mathrm{HO}}_{2}^{\bullet }= {\mathrm{Ce}}^{3+}+{\mathrm{H}}^{+}+ {\mathrm{O}}_{2}$$

2 $${\mathrm{Fe}}^{3+}+{\mathrm{HO}}_{2}^{\bullet }= {\mathrm{Fe}}^{2+}+{\mathrm{H}}^{+}+ {\mathrm{O}}_{2}$$

As shown in Fig. 2A and B, O2 is influenced to be specially adsorbed by the flawed sites of nanomaterials. Accordingly, adsorbed O_2_ is converted into O_2_^•−^ results into the concurrent oxidation of TMB whereas the Ce^4+^ and Fe^3+^ on the surface of CeFe_3_O_4_ NPs is reduced to Ce^3+^ and Fe^2+^ which will be further re-oxidized into Ce^4+^ and Fe^3+^ by generated O_2_^•−^. Therefore, chemical oxidation characteristic of CeFe_3_O_4_ NPs is accredited to both the redox switching of Ce^3+^/Ce^4+^ and Fe^2+^/Fe^3+^ and the production of O_2_^•−^ radicals (Eqs. 1 and 2) (Vinothkumar et al. 2018).

Chemical oxidation can indeed play a role in the enzyme-mimicking activities of nanoparticles (NPs). This is because the oxidation state of the NP surfaces can influence their enzyme-mimicking properties. We evaluated the activity of the oxidase enzyme using the oxidase inhibitor tetramethyl-p-phenylenediamine dihydrochloride (TMPD). The observed activities were nearly identical to those recorded in the absence of the oxidase inhibitor, as shown in Table 2. Therefore, we can conclude that the oxidase activities were primarily a result of chemical oxidation. EDS spectra analysis of CeFe_3_O_4_NPs (shown in Fig. 1C) revealed that the concentration of iron was lower than that of cerium. This resulted in a higher oxidase activity when the NPs were mixed with TMB. This improved oxidase activity was employed for lignin degradation.

### Simultaneous pretreatment and hydrolysis (SPH) process at flask- and 7.5-L fermenter scale

To ensure the sustainability of commercial-scale biofuel production from biomass, the substrate loading factor which determines the optimal substrate concentration for the conversion process is a crucial consideration. To achieve a high sugar concentration during SPH process, raw SB substrate was tested at various concentrations (5.0%, 10.0%, and 20.0% w/v). The aim was to study the effect of substrate concentration on the pretreatment of SB at the flask level before scaling up to a 7.5-L fermenter with the optimized substrate concentration.

**Fig. 3:**
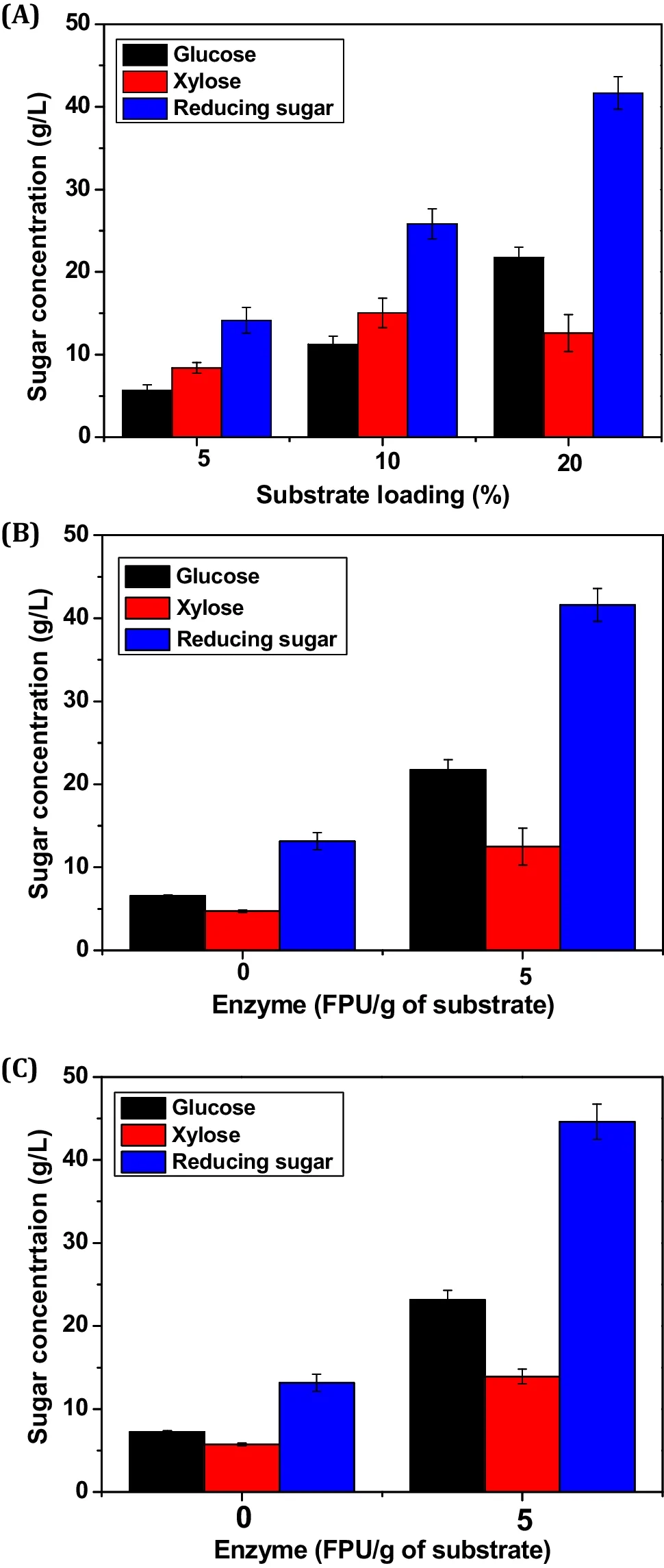
Effect of **A** substrate concentration on the release of glucose and xylose sugars during SPH process in the presence of CeFe_3_O_4_NPs (2.0 wt%), CHE supernatant (5.0 FPU/g of substrate) after 24 h; **B** CeFe_3_O_4_-NPs (2.0 wt%) on SB substrate (20%, w/v) with and without CHE supernatant at flask-level; and **C** CeFe_3_O_4_NPs (2.0 wt%) on SB substrate (20%, w/v) with and without CHE supernatant at fermenter-level

During the flask-level SPH experiments, 2.0 wt% of CeFe_3_O_4_NPs and 5.0 FPU of CHE supernatant (per gram of substrate) were added to the different substrate concentrations for optimization. As shown in Fig. 3A, the highest concentrations of glucose (21.7 ± 1.21 g/L), xylose (12.5 ± 2.22 g/L), and total reducing sugars (TRS) (41.6 ± 1.98 g/L) were achieved with 20% (w/v) SB biomass after the SPH reaction. CeFe_3_O_4_NPs were used to remove lignin during the SPH process, yielding a cellulose–hemicellulose-rich fraction. To efficiently break down these fractions, a combination of cellulase and hemicellulase enzymes along with the CeFe_3_O_4_NPs was utilized, resulting in maximum sugar release. This approach presents a promising alternative to traditional bioconversion methods. Control experiments further confirmed the cellulase–hemicellulase-mimicking activities of the CeFe_3_O_4_NPs. As depicted in Fig. 3B, the SPH process conducted without CHE supernatant, using 20% (w/v) SB biomass at the flask level, released a maximum of 6.55 ± 0.112 g/L of glucose and 4.73 ± 0.143 g/L of xylose.

As shown in Fig. 3B and C, a control experiment consisting of a flask with only CeFe_3_O_4_NPs and no enzymes released a maximum of 6.55 ± 0.112 g/L of glucose and 4.73 ± 0.143 g/L of xylose. This confirms that the CeFe3O4NPs possess cellulase- and hemicellulase-mimicking activities, capable of hydrolyzing the cellulose and hemicellulose fractions obtained during the SPS process. Additionally, a separate control experiment with only the enzymes (without nanoparticles) was conducted which showed almost negligible sugar release (data is not shown).

Based on these findings, SPH experiments were scaled up to a 7.5-L fermenter with a 5.0-L working volume as mentioned in the Materials and methods section. As shown in Fig. 3C, the sugar release profile at the fermenter scale revealed concentrations of glucose (23.1 ± 1.12 g/L), xylose (13.9 ± 0.88 g/L), and TRS (44.6 ± 2.11 g/L), which were nearly identical to the results from the flask-level experiments. The resulting hydrolysate was subsequently used for bioethanol fermentation in the same 7.5-L fermenter. Furthermore, an SPH process without CHE supernatant was performed at the fermenter scale using 20% (w/v) SB biomass. This experiment showed a sugar release profile that was similar to the flask-level results under the same conditions. During this fermenter-scale SPH process without CHE supernatant, 7.25 ± 0.132 g/L of glucose, 5.73 ± 0.153 g/L of xylose, and 13.1 ± 1.04 g/L of TRS were generated.

### Bioethanol fermentation

In this study, raw SB biomass was simultaneously pretreated and saccharified using CeFe_3_O_4_NPs and cellulase/hemicellulase enzyme (CHE) supernatant to generate hydrolysate containing glucose and xylose sugars. In addition, CeFe_3_O_4_NPs were removed magnetically from the hydrolysate obtained after SPH, but a small amount of CeFe_3_O_4_NPs (~ 5–10%) still remained attached to the minute SB biomass particles in the hydrolysate after filtration. Further, this hydrolysate was used as a carbon source by a wild-type yeast strain, *Saccharomyces cerevisiae* for bioethanol fermentation.

**Fig. 4:**
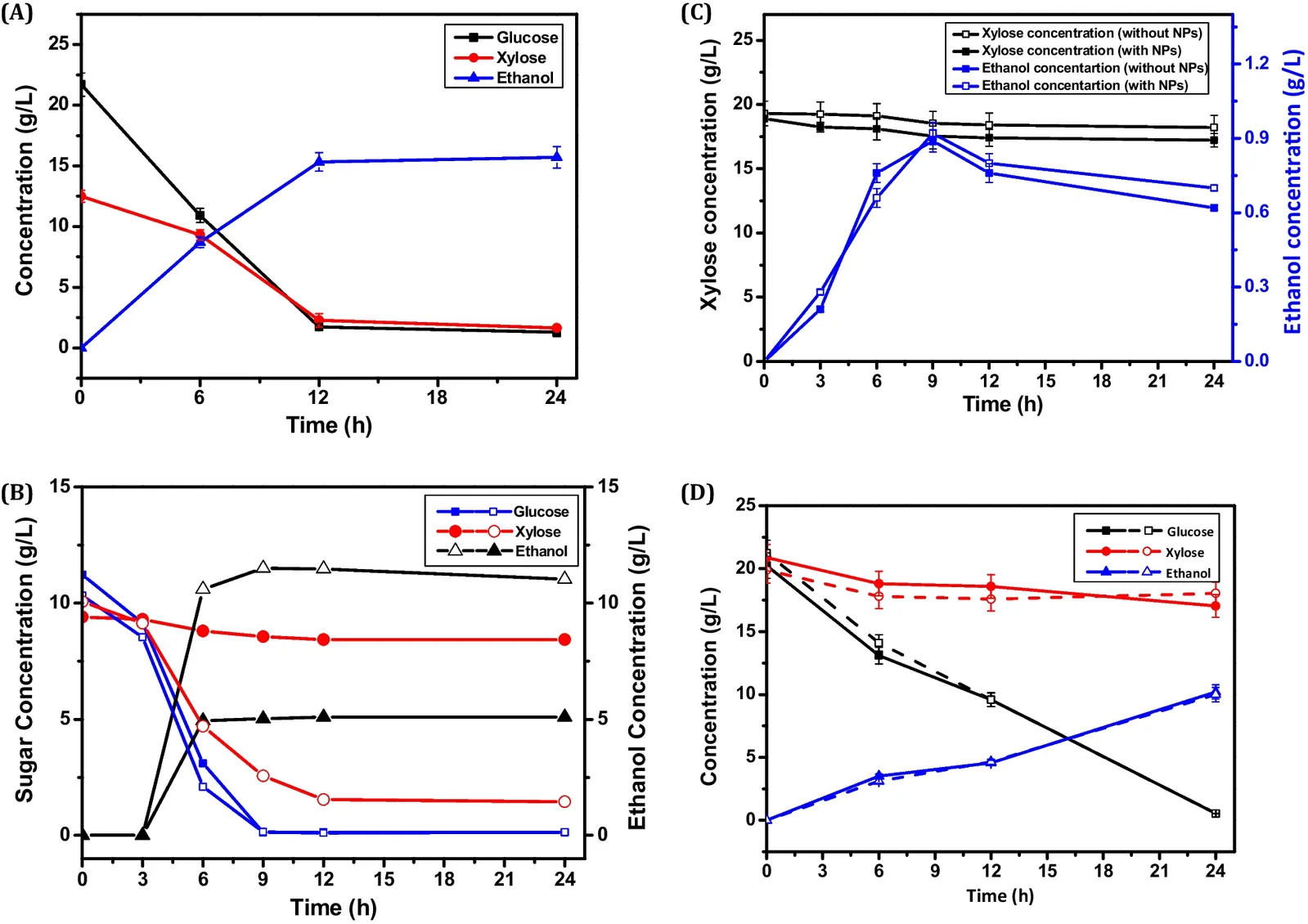
Profile of ethanol fermentation at fermenter scale using **A** SB (20% w/v) hydrlysate generated after SPH process; **B** laccase-treated SB without NPs (closed symbols) and CeFe_3_O_4_ NPs-treated SB biomass (open symbols) as carbon source; **C** pure xylose sugar (20 g/L) as carbon source with NPs and without NPs; and **D** mixture of pure glucose and xylose sugars (20 g/L) with NPs (open symbols) and without NPs (closed symbols) by *Saccharomyces cerevisiae* strain

Initially, bioethanol fermentation conditions were optimized at flask-level by using *S. cerevisiae* as mentioned in the Materials and method section. The maximum release of glucose (21.7 ± 1.21 g/L) and xylose (12.5 ± 2.22 g/L) was achieved using 20% (w/v) SB biomass after SPH reaction which has been further converted into15.7 ± 1.12 g/L of bioethanol with 1.30 g/L/h of productivity. Further, using the same conditions, bioethanol fermentation was carried out at 7.5-L fermenter-scale with working volume of 5.0 L. As shown in Fig. 3C, at fermenter-scale, the maximum 23.1 ± 1.12 g/L of glucose and 13.9 ± 0.88 g/L of xylose was obtained. Further, these sugars were utilized by the yeast strain for ethanol fermentation in a 7.5-L fermenter which generated 17.3 ± 0.98 g/L of ethanol by utilizing both glucose and xylose sugars completely as shown in Fig. 4. Microbial strains have difficulty in consuming both pentose and hexose sugars simultaneously. They start using xylose only after glucose depletion, which leads to delayed and often incomplete consumption of secondary sugars (Chiang et al. 2013). Our current study suggests that the yeast strain consumed approximately 15–20% of glucose at the beginning of fermentation and then slowly began to utilize xylose within 6 h of fermentation. The yeast strain gradually began to utilize xylose after some time, as evidenced by Fig. 4A. After 12 h of fermentation, the strain had consumed almost all sugars in the hydrolysate. This resulted in the production of 17.3 ± 0.98 g/L of ethanol from 23.1 ± 1.12 g/L of glucose and 13.9 ± 0.88 g/L of xylose with the productivity of 1.44 g/L/h. The maximum concentration of ethanol was obtained within 12 h of fermentation, with an increase in optical density from 0.1 to 8.5–9.8 in case of both flask- and fermenter-scale as depicted in Fig. S3. The study revealed that *S. cerevisiae* strain, which cannot utilize xylose, was able to effectively use both xylose and glucose to produce ethanol. HPLC chromatogram confirms the almost complete utilization of xylose (RT:9.81) along with glucose (RT:8.12) after 12 h of fermentation as depicted in Fig. S4.Furthermore, we performed ethanol fermentation studies comparing laccase-treated SB (control, without NPs) with NPs-treated SB biomass substrate (test, with NPs) as shown in Fig. 4B. The influence of NPs was particularly evident in the test experiment, where xylose was fully utilized within 12 h of fermentation, demonstrating a similar rate of glucose utilization. In contrast, the control experiment exhibited lower xylose utilization and a sequential utilization pattern with glucose. Various studies have been reported for ethanol fermentation using different substrates under varied conditions as displayed in Table 3. These results suggest that the physical and chemical properties of SB biomass enhanced xylose utilization in the presence of NPs.

**Table 3:** Comparison of bioethanol fermentation studies from different lignocellulosic substrates using various pre-treatment methods, microorganisms, and process modes

Lignocellulosic substrate	Pretreatment method	Microorganism	Mode	Bioethanol yield (g/g)	Reference
Oil palm empty fruit brances	Two-step pretreatment with 0.2 M H_2_SO_4_ at 121 °C	*K. marxinus*	SHF, batch	0.258	(Sukhang et al. 2020)
Corncob residue	KOH pretreatment	*S. cerevisiae* TC-5	SSF, Fed batch	0.281	(Boonchuay et al. 2021)
Corn cobs	2% NaOH at 120 °C for 15 min; solid-to-liquid ratio of 1:5 (w/v)	*S. cerevisiae* YI13	CBP, batch	0.1	(Davison et al. 2019)
Brewers spent grains	Dried and ground	*Aspergillus oryzae* and *S. cerevisiae*	CBP, batch	-	(Wilkinson et al. 2017)
Sugarcane bagasse	5% NaOH·H_2_O_2_, 25 °C, 24 h	*S. cerevisiae*	SSF, batch	0.101	(Raina et al. 2024)
Sugarcane bagasse	Densifying lignocellulosic biomass with chemicals followed by autoclave (DLCA)	*S. cerevisiae*	SSCF, batch	0.234	(Shen et al. 2022)
Sugarcane bagasse	Autohydrolysis, 200 °C, 10 min	Thermophilic *S. cerevisiae*	SSF, batch	0.66	(Zhang et al. 2020)
Sugarcane bagasse	Sequential acid–alkali (DAB) pretreatment using H_2_SO_4_ (3 vol%) and NaOH (5% wt.) with solid biomass in autoclave	*Kluyveromyces marxianus* JKH	SSF, Fed batch	0.260	(Hemansi and Saini 2023)
Sugarcane bagasse	Np separate pretreatment	*S. cerevisiae*	SPH	0.471	This work
Corn cob	Pretreatment with Ce-based nanomaterials	*S. cerevisiae*	SPS	0.510	(Kim et al. 2023)

In order to assess the impact of NPs on the utilization of pure xylose, we conducted experiments with and without NPs at a xylose concentration of 20 g/L in a 7.0-L fermenter. The findings, depicted in Fig. 4C indicated that NPs did not significantly affect xylose utilization in the absence of glucose. The utilization of a combination of pure glucose and xylose sugars at a concentration of approximately 20 g/L was employed as a carbon source for ethanol fermentation. The strain effectively consumed all available glucose, with minimal consumption of xylose, resulting in the production of 10.31 g/L of ethanol under both the presence and absence of NPs, as illustrated in Fig. 4D. The effect of NPs on the utilization of pure xylose sugars was found to be negligible. However, xylose sugars in SB hydrolysate were almost entirely consumed, suggesting a possible combined influence of NPs and physical and chemical properties of SB substrate. Additionally, CeFe_3_O_4_NPs may activate genes associated with xylose utilization, potentially leading to more efficient xylose utilization by the yeast strain. This research provides new insights into the role of CeFe_3_O_4_NPs in regulating key genes responsible for xylose utilization.

### Stimulation of specific xylose-utilizing genes due to CeFe_3_O_4_ NPs

**Fig. 5:**
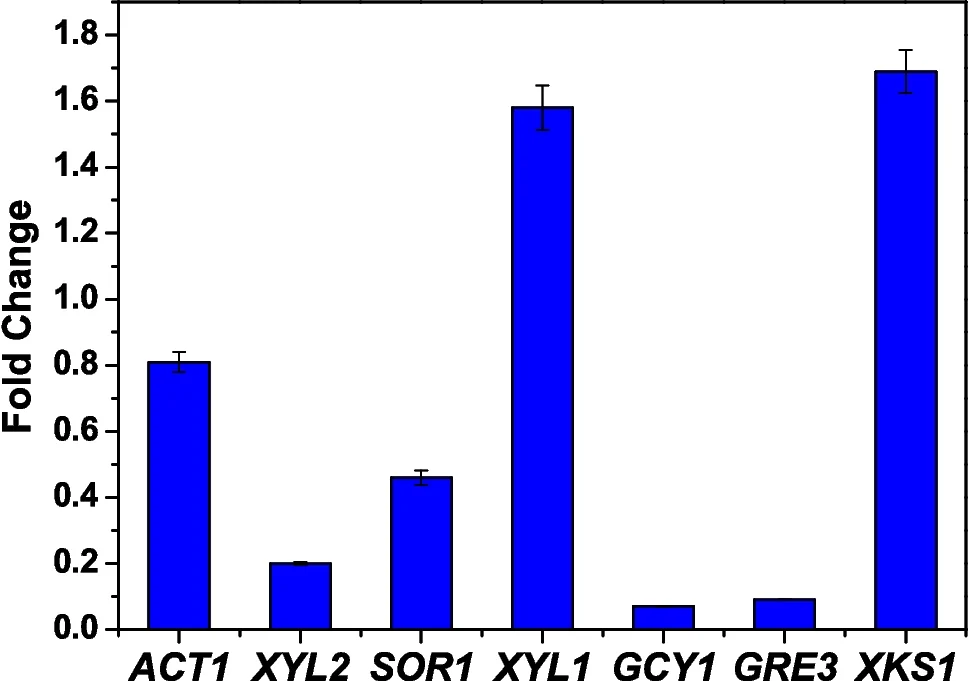
Gene expression studies of chosen genes (ACT1, XYL2, SOR1, XYL1, GCY1, GRE3, and XKS1) invloved in xylsoe conversion in *S. cerevisiae* when grown using NP-treated SB substrate as compared to control using qRT-PCR analysis

The mechanism of CeFe_3_O_4_ NPs in the expression of xylose-fermenting genes, which contribute to enhanced ethanol production from xylose and glucose present in sugarcane bagasse (SB) hydrolysate during fermentation, has been examined using quantitative real-time PCR (qRT-PCR). For this study, RNA samples were isolated from *Saccharomyces cerevisiae* grown in fermentation media containing SB hydrolysate with and without NPs. The purity of RNS samples was checked on 2.0% agarose gel as shown in Fig. S5. These samples were amplified using gene-specific primers for xylose-utilizing genes (see Table S1). The detailed information about annotation and the role of specific genes involved in xylose fermentation has been provided in Table S4. The qRT-PCR results indicated the expression levels of the selected genes: XKS1, GRE3, XYL1, SOR1, XYL2, and GCY1, which were approximately 1.72, 0.10, 1.65, 0.50, 0.22, and 0.08, respectively. Notably, as shown in Fig. 5, XKS1 and XYL1 exhibited significant increases in expression, with fold changes of 1.72 and 1.65, respectively, compared to the other genes in the presence of NPs. This study confirmed that these nanoparticles effectively enhance the yeast strain’s ability to utilize xylose at the genetic level.

#### Lignin degradation profile of SB substrate by GC–MS analysis

LCB materials contain lignin as one of the stiff components which consisted of several functional groups particularly phenolic, hydroxyl, and carboxyl groups (Katahira et al. 2018). The occurrence of functional groups improves lignin reactivity and releases lignin in intact form which can be further used for various applications (Mastrolitti et al. 2021). As shown in Table S5, the phenolic content of samples collected at different time intervals during SPH was assessed using Folin–Ciocalteau method. The maximum content of phenolic compounds was noticed at 9 h (0.331 ± 0.0032 mg/mL), and further it was constant/slightly decreased after 9 h.

To examine the released compounds after delignification of SB substrate during SPH process because of CeFe_3_O_4_NPs, GC–MS analysis has been performed. Oxidase enzymes play a substantial role in the delignification of biomass substrates in the presence of O_2_, and CeFe_3_O_4_-NPs exhibited strong chemical oxidation activity as shown in Table 2. The electron loss during oxidation of lignin generates free radicals from SB substrate which endure breaking of linkages (e.g., C–C chain), ultimately degrading lignin into monomeric aromatics and alcohol (Su et al. 2018). CeFe_3_O_4_-NPs delignified raw SB materials which produced several products at various time interval of SPH process, containing alkanes, acids, aromatics, alcohols, phenolic compounds, aldehydes, etc., as per GC–MS analysis data (Table 4). GC–MS analysis of oxidase-mimicking NP-treated SB demonstrated that new compounds were generated with increasing SPH process time, whereas other compounds were observed in the early phase of SPH reaction. Among the different products generated, some significant compounds obtained after lignin degradation have been listed in Table 4. Various aromatic alcohol compounds, phenolic, and other acid compounds such as butyl cinnamate (RT: 2.25), propanoic acid (RT: 4.712), and malic acid (RT: 10.76) were also identified. The profile of obtained compounds after lignin degradation in this study was corroborated well with the reported studies of microbial degradation of kraft lignin (Hernández et al. 2001). Some of the detected compounds in GC–MS analysis were similar as previously reported studies for lignin degradation products such as aromatic aldehydes (e.g., 3-Bromo-5-ethoxy-4-hydroxybenzaldehyde) generated from alcohol precursors upon oxidation by oxidase/laccase enzymes were observed. GC–MS profile of pretreated wheat straw and castor oil beans using laccase enzymes showed the generation of similar aldehydes (Heap et al. 2014; Mukhopadhyay and Banerjee 2015). Numerous other carboxylic acids, such as 3,5-di-tert-butyl-4-hydroxyphenyl propionic acid, 2-ethyl-3-hydroxypropionic acid, cinnamic acid, and 2-amino-alpha-(2-chlorophenyl) cinnamic acid—belonging to phenylpropanoid compounds, which play a vital role in connecting carbohydrate and lignin—were generated during the SPH process. In addition, quinoline, an aromatic compound, was detected (Christopher et al. 2014) (Christopher et al. 2014). Table 4 represents the compounds generated during SPH process at different time intervals along with their retention time. Compounds such as acetic acid and gluconic acid were also found to be present in the sample which can be attributed to hemicellulase enzymes added during SPH process. Hemicellulase causes deacetylation hemicellulose-associated acetyl groups which might have produced these compounds (Moreno et al. 2016) (Moreno et al. 2016).

**Table 4:** Total product profile of SB at different time intervals during SPH process

Identified compounds	0 h	3rd h	6th h	12th h	Retention time (min)	Relative composition (%)
Butyl cinnamate	−	−	+	−	2.250	2.61
4-hydroxybutanoic acid	−	−	−	+	2.538	1.08
Propanoic acid	−	+	+	+	4.712	
Butanoic acid	−	+	+	+	5.960	1.18
Acetic acid	−	+	+	+	5.974	0.07
1-phenylpropanol	−	−	−	+	7.142	8.47
Phenol	−	−	+	-	7.491	0.21
Benzoic acid	−	+	+	+	8.011	0.10
Cinnamic acid	−	−	+	+	8.510	0.75
Quinoline	−	+	−	−	10.12	
2-Amino-alpha-[2chlorophenyl] cinnamic acid	−	+	−	−	10.24	0.43
2-Ethyl-3-hydroxypropionic acid	−	−	+	−	10.51	0.11
Butanedioic acid	−	−	+	+	10.58	0.09
Malic acid	−	+	+	+	10.76	1.56
3,5-di-tert-Butyl-4-hydroxyphenyl propionic acid	−	+	−	−	11.28	0.12
D-Glucitol	−	+	+	+	13.01	0.31
Xylitol	−	+	+	+	13.49	3.01
β-D-Galactofuranose	−	−	+	+	14.91	1.98
D-Xylose	−	+	+	+	15.34	5.64
Gluconic acid	−	+	+	+	16.09	0.91
D-Glucose	−	+	+	+	16.22	7.33
Succinic acid	−	+	+	+	16.46	0.66
Octadecanoic acid	−	+	+	+	18.283	1.17
3-Bromo-5-ethoxy-4-hydroxybenzaldehyde	−	−	+	+	18.76	0.76
Ethyl 2-[4-chlorophenyl]−7,8-benzo cinchoninate		+	−	−	21.79	0.72
2-[2′-Phenoxathiinyl]cinchoninic acid		−	+	+	21.09	0.43
D-Arabinose	−	+	+	+	22.545	0.09

‘ + ’ sign indicates the presence of particular compound and ‘ − ’ sign indicates the absence of particular compound

### Structural characterization of untreated and treated SB sample

To compare untreated SB (before SPH) and treated SB (after SPH), we conducted characterization studies using SEM, TEM, FTIR, XRD, thermogravimetric analysis (TGA), and derivative thermogravimetric (DTG) analysis on both biomass samples. Both untreated and CeFe_3_O_4_NP-treated sugarcane bagasse (SB) were compared using SEM and FTIR to evaluate the effects of the SPH process. SEM images revealed that untreated SB (Fig. S6A) had a smooth, dense surface, while treated SB (Fig. S6B) showed a rough, fibrous, and porous structure due to the removal of the outer lignin layer by the nanoparticles. FTIR analysis confirmed these structural changes, showing a decrease in peaks corresponding to lignin (1490 and 1230 cm^−1^), hemicellulose (1710 cm^−1^), and modified cellulose (1070, 1320, and 1410 cm^−1^), indicating that the CeFe_3_O_4_NPs successfully delignified the biomass and altered its structure to release fermentable sugars as shown in Fig. S6C. The XRD analysis revealed that SPH process, mediated by CeFe_3_O_4_NPs, caused a crucial reduction in crystallinity of SB samples, decreasing its crystallinity index (CrI) from 58.61 to 49.12% (Fig. S6D). This is attributed to the breakdown of non-cellulosic components (lignin and hemicellulose) and deconstruction of the carbohydrate part, increasing the accessibility of the biomass. Concurrently, the crystallite size surprisingly increased from 2.42 to 3.21 nm (as displayed in Table S6), which is consistent with the decreased overall crystallinity and likely results from the disruption of ether linkages between lignin and carbohydrates. This pretreatment also significantly enhanced the biomass’s texture, increasing its total pore volume from 0.0021 to 0.0039 mL/g and its surface area from 1.142 to 2.042 m^2^/g as shown in Table S7. The pore size also became larger (increasing from 16.97 to 31.20 nm). These structural changes collectively enhance enzyme diffusion and accessibility to the cellulose, supporting a higher release of fermentable sugars, which is the ultimate goal of the SPH process.

Thermogravimetric analysis (TGA) was used to compare the thermal degradation of untreated and CeFe_3_O_4_NP-treated SB samples, revealing that the treatment significantly alters the thermal properties. The treated SB showed a higher percentage of weight loss compared to the untreated sample, indicating greater degradation during the SPH process. As shown in Fig. S6A and B, a shift in the primary degradation peak from 370 °C for untreated SB to 385 °C for treated SB suggests that the nanoparticles caused further degradation of the cellulose component, leading to a higher degradation temperature. These results demonstrate that the CeFe_3_O_4_NP effectively promote the thermal breakdown of the SB biomass, supporting the enhanced release of fermentable sugars.

### Biocompositional analysis of untreated and treated SB samples

LCB are potential feedstocks for producing chemicals and biofuels, which primarily depend on their carbohydrate composition (i.e., cellulose and hemicellulose). The biochemical composition of both untreated and treated SB samples was evaluated using method reported by Ayeni et al. (Ayeni et al. 2015). The weight balance of the treated and untreated SB samples is briefed in Table 5. The untreated SB biomass (8.0 g) was mainly composed of cellulose (3.42 ± 0.15 g) and hemicellulose (2.30 ± 0.11 g), in consort with lignin component (1.80 ± 0.10 g), which has to be detached to expose carbohydrate moieties. The lignin content of the treated SB was reduced by 38.33% after CeFe_3_O_4_ NPs pretreatment, and simultaneously, 36.25% and 30.40% of cellulose and hemicellulose were seemed to be dropped after SPH process. The ultimate mass of treated SB after SPH was found to be 4.95 g, indicating untreated SB conversion of 38.12%. These variations in biomass composition demonstrate the effect of CeFe_3_O_4_NPs on untreated SB during SPH process. There has been number of studies reported about various pretreatment processes impacting the biomass composition (Singhvi et al. 2021b; Rajak et al. 2021). Finally, the sugars released during hydrolysis of cellulose-hemicellulose were further used to produce bioethanol.

**Table 5:** Biocompositional analysis of untreated SB (before SPH) and treated SB (after SPH) samples

Biomass components	Untreated SB (g)	Treated SB (g)	Conversion (%)
Cellulose (42.7%)	3.42 ± 0.15	2.18 ± 0.08	36.25
Hemicellulose (28.7%)	2.30 ± 0.11	1.60 ± 0.12	30.40
Lignin (22.5%)	1.80 ± 0.10	1.11 ± 0.07	38.33
Ash (−)	0.01 ± 0.01	0.04 ± 0.01	
Extractives (−)	0.47	0.02	-
Total	8	4.95	38.12

### Recycling of CeFe_3_O_4_ NPs left after SPH process

CeFe_3_O_4_NPs exhibit oxidase activities due to chemical oxidation and magnetic properties that make them easily separable from hydrolyzed broth. The study investigated the recovery and reusability of CeFe_3_O_4_NPs in the SPH process. Recovered NPs were obtained from broth after SPH process containing 20% (w/v) untreated SB substrate. After 24 h of SPH experiment, the obtained supernatant was analyzed for glucose, xylose, and TRS concentrations. As shown in Fig. S8, TRS after first cycle was 79%, which decreased in the second recycling to 72%. The sugar release profiles were investigated during the recycling of used CeFe_3_O_4_NPs. The first cycle yielded 21.7 g/L of glucose and 12.5 g/L of xylose, while the second and third cycles produced 13.2 g/L and 12.7 g/L of glucose and 11.9 g/L and 10.7 g/L of xylose, respectively (Fig. S8). Overall sugar concentrations were reduced in succeeding cycles using reused CeFe_3_O_4_ NPs. However, reused NPs maintained around 60–65% efficacy even after the third cycle. The achieved results substantiate the prospect of reusing NPs in converting biomass into sugars. This experiment indicated useful catalytic abilities even after reusing CeFe_3_O_4_NPs for delignifying LCB substrates. The magnetic properties of CeFe_3_O_4_NPs allowed for easy separation from the hydrolysate broth after SPH process.

## Discussion

Lignocellulosic biomass (LCB) is a renewable, non-edible source of fermentable carbon. However, its conversion into biofuels is significantly hindered by complex lignin–carbohydrate linkages. Traditional physical, chemical, and biological pretreatment methods often suffer from drawbacks such as toxicity, high costs, and limited efficiency. To address these limitations, eco-friendly and sustainable alternatives are urgently required. Recent advancements in nanotechnology particularly the emergence of enzyme-mimicking nanoparticles known as “nanozymes” offer a promising route for efficient and green biomass pretreatment.

In this study, oxidase-mimicking cerium-iron oxide nanoparticles (CeFe_3_O_4_NPs) were synthesized and characterized using various analytical techniques (as detailed in the Results section). The synthesis of CeFe_3_O_4_NPs via a modified method was confirmed by various techniques, which showed agglomerated, sphere-shaped particles measuring 11–16 nm and a negative surface charge that contributes to stability. Elemental analysis indicated a higher concentration of cerium than iron, while XRD confirmed a single-phase inverse spinel structure similar to pure Fe_3_O_4_, demonstrating that cerium doping did not alter the core structure. DLS analysis determined that synthesized CeFe_3_O_4_ nanoparticles have a zeta potential of − 15.0 ± 3.92 mV, indicating a stable, net negative surface charge. This result aligns with other published research on cerium-based nanomaterials (Zheng et al. 2018; Aponte et al. 2020). XRD analysis results indicate that the modification of magnetite with rare elements did not change the crystal structure of Fe_3_O_4_, thus the existence of Ce^3+^ in the lattice kept the structure unchanged (Wechsler et al. 1984). The XRD peaks provide essential information about the location of the dopant in the crystal lattice. These peaks are consistent with the standard pattern of cubic inverse spinel structure. The results indicate that the modification of magnetite with rare elements did not change the crystal structure of Fe_3_O_4_; thus, the existence of Ce^3+^ in the lattice kept the structure unchanged (Wechsler et al. 1984). It also specifies that agglomeration persists due to the magnetic property of Fe_3_O_4_.

CeFe_3_O_4_NPs exhibited both cellulase–hemicellulase- and oxidase-mimicking activities, with the oxidase-like activity being notably higher than previously synthesized nanoparticles (due to increased cerium concentration. This oxidase activity occurs via chemical oxidation involving redox switching and superoxide radical generation. The enzyme-mimicking activities of these nanoparticles were evaluated for their potential to degrade lignin in lignocellulosic materials, eliminating the need for a separate pretreatment step. Oxidase enzymes catalyze the oxidation of specific substrates, using molecular oxygen as an electron acceptor to generate oxidized products along with H_2_O or H_2_O_2_. Similarly, CeFe_3_O_4_ NPs catalyze substrate oxidation via electron transfer mechanisms (Grebel et al. 2016), showing oxidase-like activity under ambient conditions (Lang et al. 2014; Xiong et al. 2015; Peng et al. 2018; Jin et al. 2020). Cerium-based nanomaterials, due to their Ce^3+^/Ce^4+^ redox cycling, effectively catalyze the oxidation of TMB (3,3′,5,5′-tetramethyLCBenzidine), mimicking natural oxidases with similar kinetic behavior (Cheng et al. 2016; Zhou et al. 2017). Several studies have indicated that cerium-based nanoparticles exhibit oxidase-mimicking behavior, as observed in the present investigation (Jansman and Hosta-Rigau 2019; Kim et al. 2023).

In addition to oxidase activity, the synthesized CeFe_3_O_4_ NPs exhibited cellulase- and hemicellulase-mimicking activities, enabling their use in the simultaneous pretreatment and hydrolysis (SPH) of raw sugarcane bagasse (SB). During this process, the NPs facilitated delignification, enriching the cellulose and hemicellulose fractions, while also enabling concurrent enzymatic saccharification. To enhance sugar release, in-house produced cellulase–hemicellulase (CHE) enzymes from *Penicillium janthinellum* were also employed. As the use of carbon sources accounts for approximately 50% of enzyme production costs (Toor et al. 2020), this study utilized SB biomass a cheap, abundant lignocellulosic waste as a cost-effective fermentation medium for enzyme production. This approach offers nutritional and rheological benefits, promoting fungal growth and enhanced CHE production. Notably, only a few studies have reported such high CHE activity using SB as a sole carbon source at the fermenter scale. Recently, the maximum cellulase enzyme activities including endoglucanase (1.38 U/mL) and β-glucosidase (0.55 U/mL), after the 5th and 6th days of fermentation, respectively, using SB as substrate have been reported (de Castro Coêlho et al. 2021). Rocha et al. (Rocha et al. 2016) reported highest CMCase (1.0 ± 0.1 U/mL), FPase (0.06 ± 0.01 U/mL), and β-glucosidase (0.4 ± 0.1 U/mL) activities by *Penicillium* sp. FSDE15 after 120 h of fermentation, using sugarcane straw substrate which are lesser than those obtained in the present study. Consequently, the complete set of CHE cocktails secreted by *P. janthinellum* under submerged conditions were further employed for releasing fermentable sugars from raw SB substrate.

The combined action of CeFe_3_O_4_ NPs and CHE enzymes during SPH resulted in maximal sugar release, with glucose and xylose yields reaching 21.7 ± 1.21 g/L and 12.5 ± 2.22 g/L, respectively. It has been reported that very few studies on the simultaneous pretreatment and hydrolysis of biomass substrates have demonstrated significant sugar release (Singhvi et al. 2021b; Rajak et al. 2021; Kim et al. 2023). Table 3 represents the comparative data of ethanol fermentation using different LCB substrates including SB through various approaches. In this study, sugar release occurred even in the absence of enzymes, underscoring the catalytic hydrolytic ability of CeFe_3_O_4_ NPs. The optimal temperature for the SPH process was 50 °C wherein cellulase and hemicellulase enzymes retained 70–80% activity (Brunecky et al. 2014; Peng et al. 2015). At this temperature, CeFe_3_O_4_ NPs showed enhanced interaction and adsorption to biomass surfaces, improving oxidation due to cerium ion doping (Nassar et al. 2019).

Following saccharification, CeFe_3_O_4_ NPs were removed from the hydrolysate via magnetic separation. Although minor residues remained, the hydrolysate rich in glucose and xylose was utilized for bioethanol fermentation using *Saccharomyces cerevisiae*. Generally, yeast strains can efficiently ferment hexose sugars; however, they cannot metabolize pentoses (which represent 40–45% of LCB sugars), presenting a major bottleneck (Kumar et al. 2018). Despite genetic enhancements (Dien et al. 2003; Fernández-Sandoval et al. 2012), improved xylose metabolism in *S. cerevisiae* remains elusive due to carbon catabolite repression (CCR), which favors glucose uptake (Görke and Stülke 2008; Vinuselvi et al. 2012). However, this study demonstrated that the yeast strain used could simultaneously co-ferment glucose and xylose, indicating a reduction in CCR, an uncommon but commercially valuable trait. This dual sugar utilization significantly improved ethanol yield and productivity. It is possible that the presence of CeFe_3_O_4_NPs in the hydrolysate might have activated genes responsible for the consumption of xylose in *S. cerevisiae* strain. Studies have shown that exposure to NPs can cause alterations in genomic and proteomic profiles, which suggests that the NPs can modulate cellular adaptation to a new environment. Earlier studies have shown that bacterial cells in the presence of silver nanoparticles (NPs) and ions exhibited up-and downregulation of genes (Pelletier et al. 2010; McQuillan and Shaw 2014). Similarly, the presence of CeFe_3_O_4_NPs may have caused differential gene expression in *S. cerevisiae* strain, particularly in genes responsible for xylose utilization (Niazi et al. 2011). This study confirmed the action of NPs phenotypically in efficiently utilizing xylose by the yeast strain. To gain a better understanding of how wild-type *S. cerevisiae* consumes xylose when exposed to CeFe_3_O_4_NPs, it would be helpful to conduct further investigations into the expression levels of specific xylose-utilizing genes. As displayed in Table 3, the comparative analysis of all the reported studies with the current work showed the higher ethanol yield in the presence of NPs due to co-consumption of xylose along with glucose from SB substrate.

To investigate the underlying mechanism, qRT-PCR analysis was conducted to evaluate gene expression related to xylose fermentation in the presence of CeFe_3_O_4_ NPs. Results confirmed that NPs enhanced the expression of xylose-fermenting genes, enabling more efficient pentose utilization and ethanol production from SB hydrolysate. In addition to bioethanol, GC–MS analysis of degradation products post-SPH revealed the presence of low molecular weight acids, alcohols, esters, and aldehyde compounds with commercial value in the fragrance and aroma industries (Pandey and Kim 2011). This confirms the NPs’ role in cleaving complex lignin structures (Rich et al. 2016; Feng et al. 2019).

To support our findings, we conducted physical and characterization studies to compare the modifications in the treated SB samples with the untreated samples. Scanning electron microscopy (SEM) and Fourier-transform infrared spectroscopy (FTIR) were used to analyze the structural changes in sugar bagasse (SB) samples after treatment with CeFe_3_O_4_ nanoparticles during the SPH process. SEM images showed that treated SB developed a porous, loosened, and fibrous surface with visible holes and clefts, contrasting sharply with the dense and intact surface of untreated samples (Fig. S6A and B) (Zhao et al. 2010; Lin et al. 2020). This morphology change suggests strong interaction between the NPs and biomass, leading to the removal of the outer lignin layer. FTIR analysis further supported these findings by identifying changes in functional groups (Fig. S6C). A decrease in the ester group peak at 1710 cm^−1^ indicated hemicellulose degradation, while reduced peaks at 1490 cm^−1^ and 1230 cm^−1^ corresponded to lignin removal as per reported studies (Lin et al. 2020; Rajak et al. 2021; Kim et al. 2023). Additionally, alterations in peaks at 1070, 1320, and 1410 cm^−1^ revealed modifications to the cellulose component (Charis et al. 2020). These results confirm that the CeFe_3_O_4_ NPs effectively delignify the biomass and modify cellulose and hemicellulose, thereby aiding in the release of fermentable sugars (Althuri et al. 2017; Singhvi et al. 2021b). XRD analysis of treated and untreated sugar bagasse (SB) showed that the SPH process using CeFe_3_O_4_ nanoparticles decreased the crystallinity index (CrI) from 58.61 to 49.12% while increasing crystallite size from 2.42 to 3.21 nm. This reduction in crystallinity, which enhances biomass accessibility, facilitates greater sugar release, a finding supported by observed higher sugar yields after the SPH process. These findings are well corroborated with previously reported research studies (Zhao et al. 2010; Lin et al. 2020).

During the SPH process, CeFe_3_O_4_ NPs significantly enhanced the porosity of sugar bagasse (SB) biomass, increasing the total pore volume and surface area while reducing the pore size from 31.20 to 16.97 nm (Kwok et al. 2017; Li et al. 2018). This improved porosity, caused by the degradation of lignin and hemicellulose, facilitates enzyme diffusion and loading, thereby boosting the release of fermentable sugars (Guo and Catchmark 2012; Meng et al. 2015). TGA further revealed that the CeFe_3_O_4_ NPs treatment altered the biomass’s thermal degradation profile. The treated SB showed an increase in the cellulose degradation temperature, shifting the peak from 370 to 385 °C compared to untreated SB, which is consistent with some previous studies (Najafi et al. 2024). This thermal behavior confirms the modification of the biomass’s thermal stability.

Following ethanol fermentation, the magnetic properties of CeFe_3_O_4_ NPs not only enable their effortless recovery but also support their reuse in multiple subsequent experiments. This finding underscores the potential of integrating nanozymes with minimal enzymatic input into the SPH process. This approach presents a promising and sustainable pathway for large-scale, industrial conversion of lignocellulosic biomass into biofuels and a range of other valuable biochemicals.

## Supplementary Information

Below is the link to the electronic supplementary material.

ESM 1 (DOCX 2.20 MB)

## Data Availability

All data supporting the findings of this study are available within the paper and its.

## Abbreviations

GHG: Greenhouse gas
LCB: Lignocellulosic biomass
SB: Sugarcane bagasse
LCCs: Lignin–carbohydrate complexes
NPs: Nanoparticles
MoOx/CNT: Molybdenum oxide carbon nanotubes
CHE: Cellulase–hemicellulase enzymes
SPH: Simultaneous pretreatment and hydrolysis
TEM: Transmission electron microscopy
SEM: Scanning electron microscopy
EDS: Energy-dispersive X-ray spectroscopy
FTIR: Fourier transform infrared
PDA: Potato dextrose agar
BM: Basal medium
DO: Dissolved oxygen
FPU: Filter paper unit
TMB: 3,3′,5,5′-Tetramethylbenzidine
HPLC: High-performance liquid chromatography
LSB: Laccase-treated SB
RSB: Raw SB
