# Supplementary material for: Synergistic effect of Ce-based nanocatalysts in the pretreatment and saccharification of raw lignocellulosic biomass: an advancement in bioethanol production
Source: Appl Microbiol Biotechnol. 2025 Dec 8;109:254. doi: 10.1007/s00253-025-13627-7 (PMC12689739; doi:10.1007/s00253-025-13627-7)
Supplement: Supplementary file 1 [file 253_2025_13627_MOESM1_ESM.docx]

**Supplementary information for**

**Synergistic effect of Ce-based nanocatalysts in the pretreatment and saccharification of raw lignocellulosic biomass : an advancement in bioethanol productiona**

**Mamata S. Singhvi^1^*, Chinmay Hate^1^, Beom Soo Kim^2^**

**^1^Department of Biotechnology (with Jointly Merged Institute of Bioinformatics and Biotechnology), Savitribai Phule Pune University, Pune 411007, India**

**^2^Department of Chemical Engineering, Chungbuk National University, Cheongju,**

**Chungbuk 28644, Republic of Korea**

**Corresponding Author*:**

**Mamata S. Singhvi**

**Department of Biotechnology (with Jointly Merged Institute of Bioinformatics and Biotechnology),**

**Savitribai Phule Pune University,**

**Pune 411007, India**

**E-mail: mamatasinghvi@gmail.com**

**Fig.S1.** XRD analysis of synthesized CeFe_3_O_4_ NPs

**Fig.S2.** Magnetic property of synthesized CeFe_3_O_4_NPs

**Fig. S3.** Growth profile of *Saccharomyces cerevisiae* during ethanol fermentation using SB (20% w/v ) hydrlysate generated after SPH process at flask- and fermenter-scale at different time inetrvals.

**Fig.S4.** HPLC chromatogram of raw SB hydrolysate samples treated with NPs obtained after 12h of ethanol fermentation by *S. cerevisiae* strain. This chromatogram exhibits the utilization of xylose after 12h fermentation in presence of CeFe_3_O_4_ NPs.

1

2

**Fig.S5.** Purity check of extracted total RNA samples on agarose gel (2.0%). Lanes 1 & 2 correspond to RNA samples extracted from *S. cerevisiae* cells grown using Control (sample 1, without NPs) and Treated (Sample 2, with NPs) SB samples respectively.

**(A)**

**(B)**

**(D)**

**(C)**

**Fig. S6.** Characterization of untreated and treated SB (after SPH) samples using(A) SEM, (B) TEM, (C) FTIR and (D) XRD

**(A)**

**(A)**

**(B)**

**Fig.S7.** Characterization of : A) untreated and B) treated SB biomass samples using TGA analysis.

**Fig. S8. (**A)Recycling of recovered CeFe3O4NPs and their effect on glucose, xylose, and TRS release during SPH process.

**Table S1**

Primers used for qRT-PCR analysis of specific xylose utilizing gene expression in *Saccharomyces cerevisiae*

| Gene name | Primers (forward, reverse) |
| --- | --- |
| XYL2 | TGGTGCTCAAGCCTGCATTA  TCGCTGTAGTCTCCCTGACA |
| SOR1 | AGTCGGCGATATTGCCATCG  GTCTTGACTACCTCTCCACCATG |
| XYL1 | GCCATCATTGGGACAGGTACTAG GTGCCTTCCCAACTTCTGGAT |
| GCY1 | GACTGGTAAAACTAAGGCCGT  ACAATCATACGAGAAACACGCA |
| GRE3 | TCCTCAATCATTCATTGAGAGT  TCTCAATGAATGATTGAGGAG |
| Xylulokinase  (XKS1) | TTCCTCTGGGGGAGATCGTT  TCTCTGTTGTGAGCTTGCGT |
| ACT 1 | ATTATATGTTTAGAGGTTGCTGCTTTGG CAATTCGTTGTAGAAGGTATGATGCC |
|  |  |

**Table S2. Zeta Potential analysis of CeFe_3_O_4_NPs**

**Table S3**

Cellulase and hemicellulase enzyme production by *P. Janthinellum* NCIM 1171 under submerged fermentation conditions at flask level using different substrates after 6 days of fermentation

| **Substrates used** | | **Enzyme activities**  **(IU/mL)** | | | | | | |  | |  | |
| --- | --- | --- | --- | --- | --- | --- | --- | --- | --- | --- | --- | --- |
|  | | **Cellulase** | | | | | |  | | **Hemicellulase** | | |
|  | **Exoglucanase** | | **Endoglucanase** | | **β-glucosidase** |  | **Xylanase** | | | | |  |
| **WB** | 0.132 ±0.009 | | 1.861±0.010 | 0.982±0.011 | |  | 0.987±0.041 | | | | |  |
| **CC** | 0.234 ±0.014 | | 2.942±0.155 | 0.864±0.051 | |  | 1.560±0.062 | | | | |  |
| **SB** | 0.427±0.018 | | 3.604±0.201 | 3.783±0.152 | |  | 2.268±0.123 | | | | |  |
| **SH** | 0.281 ±0.021 | | 3.107±0.201 | 0.5443±0.002 | |  | 0.991±0.023 | | | | |  |

*Fermentation experiments were carried out at 30 ^o^C under submerged conditions in fermentation medium containing various biomass substartes (2.5%) and cellulose (1.0%) substrates. The standard deviation values represented in the table are derived from the experiment performed in triplicates.*

**Table S4**

Annotation and description of specific genes involved in xylose fermentation.

| Gene symbol | Annotation | Description |
| --- | --- | --- |
| XKS1 | Xylulokinase | converts D-xylulose and ATP to xylulose 5-phosphate and ADP |
| GRE3 | Aldose reductase | involved in methylglyoxal |
| XYL1 | Uncharacterized oxidoreductase YJR096W | Xylose and arabinose reductase |
| SOR1 | Sorbitol dehydrogenase | protein sequence is 99% identical to the Sor2p sorbitol dehydrogenase |
| XYL2 | D-xylulose reductase | Xylitol dehydrogenase |
| GCY1 | Glycerol 2-dehydrogenase (NADP(+)) | Glycerol dehydrogenase |

**Table S5** Analysis of total phenolic content obtained at various time intervals during SPH process

| **Time (h)** | **Phenolic contents (mg/mL)** |
| --- | --- |
| 3 | 0.348 ± 0.011 |
| 6 | 0.441 ± 0.024 |
| 12 | 0.469 ± 0.011 |
| 24 | 0.411 ± 0.012 |

**Table S6** Investigation of crystallinity index (CrI) and crystallite sizes (nm) of untreated SB

(before SPH) and treated SB (after SPH) samples

| **Samples** | **Crystallinity index**  **(crI) (%)** | **Relative decrease in crystallinity (%)** | **Crystallite size**  **(nm)** |
| --- | --- | --- | --- |
| Untreated SB | 58.61 | - | 2.42 |
| Treated SB | 49.12 | 16.19 | 3.21 |

**Table S7** Porosity of untreated and treated sugarcane bagasse biomass samples.

| **Biomass solids** | **Surface area (m²/g)** | **Pore volume (mL/g)** | **Pore diameter (nm)** |
| --- | --- | --- | --- |
| Untreated SB | 1.142 | 0.0021 | 16.97 |
| Treated SB | 2.042 | 0.0039 | 31.20 |
